# Combining Deep Learning and Hydrological Analysis for Identifying Check Dam Systems from Remote Sensing Images and DEMs in the Yellow River Basin

**DOI:** 10.3390/ijerph20054636

**Published:** 2023-03-06

**Authors:** Mengqi Li, Wen Dai, Mengtian Fan, Wei Qian, Xin Yang, Yu Tao, Chengyi Zhao

**Affiliations:** 1 School of Geographical Sciences, Nanjing University of Information Science and Technology, Nanjing 210044, China; 2 School of Remote Sensing and Geomatics Engineering, Nanjing University of Information Science and Technology, Nanjing 211800, China; 3 School of Geography, Nanjing Normal University, Nanjing 210023, China; 4 School of Geographical Information and Tourism, Chuzhou University, Chuzhou 239000, China

**Keywords:** check dam system extraction, object-based image analysis, Yellow River Basin, terrain analysis, deep learning

## Abstract

Identifying and extracting check dams is of great significance for soil and water conservation, agricultural management, and ecological assessment. In the Yellow River Basin, the check dam, as a system, generally comprises dam locations and dam-controlled areas. Previous research, however, has focused on dam-controlled areas and has not yet identified all elements of check dam systems. This paper presents a method for automatically identifying check dam systems from digital elevation model (DEM) and remote sensing images. We integrated deep learning and object-based image analysis (OBIA) methods to extract the dam-controlled area’s boundaries, and then extracted the location of the check dam using the hydrological analysis method. A case study in the Jiuyuangou watershed shows that the precision and recall of the proposed dam-controlled area extraction approach are 98.56% and 82.40%, respectively, and the F1 score value is 89.76%. The completeness of the extracted dam locations is 94.51%, and the correctness is 80.77%. The results show that the proposed method performs well in identifying check dam systems and can provide important basic data for the analysis of spatial layout optimization and soil and water loss assessment.

## 1. Introduction

**Figure 1:**
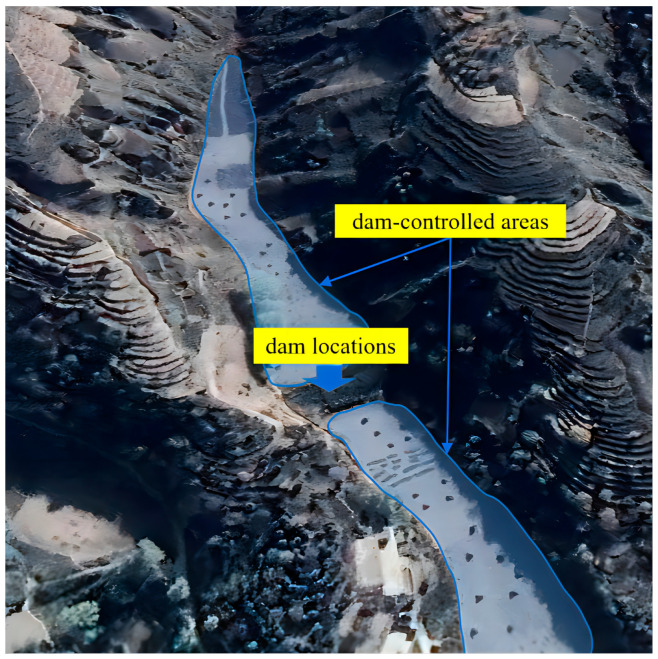
Check dam system in the Yellow River Basin.

A check dam system is a small, sometimes temporary, structure constructed across a swale, drainage ditch, or waterway to counteract erosion by reducing water flow velocity [1]. Due to the intense erosion, many check dams have been constructed in the Yellow River Basin [2]. In each watershed, large, medium, and small check dams are distributed at different levels of the gully networks, and the check dams are mostly located in the ditch. Various large, medium, and small check dams work in coordination with each other to form a tree-like structure of check dam systems. The check dam system includes the dam location and dam-controlled area (Figure 1), and different components have different functions. Check dam systems have a remarkable effect on preventing soil erosion and improving agricultural management. The dam-controlled area has rich minerals for crop cultivation, which offer another opportunity for crops in the Yellow River Basin. In order to realize the rational use of dam-controlled areas and optimize the allocation of agricultural land, obtaining accurate information about the dam-controlled areas is a prerequisite. At the same time, accurately identifying the dams’ locations is critical for analyzing the functions of check dams in preventing soil erosion and catching sediment, as well as optimizing the construction layout of check dam systems [2].

With the development of remote sensing technology, it is possible to obtain ground feature information from remote sensing data. Although manual visual interpretation and field investigation can achieve high accuracy in check dam extraction, they are inefficient and laborious and are not suitable for large-scale dam identification. IKONOS high-resolution remote sensing images were used to extract information on land use and soil and water conservation practices with GIS-driven methods [3]. There are several ways to acquire images. Using unmanned aerial vehicles (UAVs) [4] to obtain high-resolution aerial photos to determine the location of dams is flexible, short-period, and high-resolution. The pixel-based image classification had been proposed to automatically classify remote sensing images [5], but this method could lead to serious misclassification and impulse noises [6,7,8]. The OBIA [6] method is widely used in geography, which has solved the problems mentioned above by exploiting shape, spectral, and texture information in high-resolution images [9]. Wei et al. [10] quantified loess landforms using an object-based image analysis (OBIA) method and used this classification to describe the spatial variability of loess landforms. However, the OBIA method cannot detect detailed high-level features (such as semantic features between different pairs of images) [7,11].

As algorithms for processing images get better, deep learning offers alternatives and shows some superiority [12]. The U-Net model has achieved remarkable results in medical target monitoring and has been extended to the field of geoscience research. It has shown the ability of deep-learning methods to extract surface hydrological features from complex hydrological features [13]. Li et al. [14] used U-Net, random forest, and CNN models to extract the dam-controlled area, and the comparison of the results shows that U-Net was better at extracting the dam-controlled area. In recent years, buildings, water bodies, roads, and impermeable surfaces [15,16,17] are the main ground objects that deep-learning methods have been used to detect. Check dams are usually small in number and size and are difficult to identify from low-resolution or medium-resolution images. Therefore, our literature review found few studies focusing on dam location detection. Sun et al. [18] used 1 m remote sensing images and 30 m DEM to detect medium and large dams, whose sizes are larger than 50 m, using the deep-learning method, but were not able to solve the issue of extracting small dams.

This study offers insights into automatically identifying dam locations and dam-controlled areas from 0.5 m remote sensing images and 5 m DEMs and aims to realize synchronous observation over a large area. We used the Jiuyuangou watershed in the Yellow River Basin as the study area. To improve the extraction of dam-controlled areas, a methodology combining deep learning (the U-Net model) and the OBIA method [15,19] is proposed. It considerably improves the efficiency of ground-object extraction and categorization in geographic research. On the one hand, the OBIA method can effectively avoid the classification noise generated by pixel-based image analysis and reduce the homogeneity of different features and the heterogeneity of similar features [20]. On the other hand, deep learning can extract high-level features that can accurately classify semantic objects [12]. Considering the spatial relationship between the dam-controlled area and the dam location, we inferred dam locations through the hydrological analysis based on the dam-controlled area results.

## 2. Materials and Methodology

### 2.1. Study Area and Data

**Figure 2:**
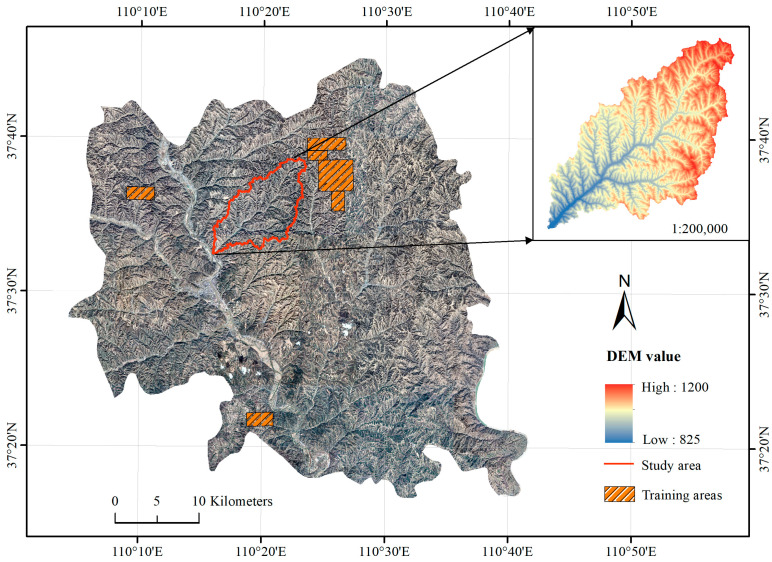
The location of the study area.

The Jiuyuangou watershed (37°33′~37°38′ N, 110°16′~110°26′ E), located in Suide County, Shaanxi Province, China, was chosen as the study area (Figure 2). The Jiuyuangou watershed as a study area is representative because many gullies developed in this region, and many check dams were constructed for soil and water conservation. The Jiuyuangou watershed, with a main gully length of 18 km, 337 branch gullies above 200 m, a gully density of 5.34 km/km^2^, and a basin elevation between 820 m and 1180 m, is a branch gully in the middle reaches of the Wuding River. The total area of the study area is 69.29 km^2^; the actual dam-controlled area is 2.50 km^2^, and the rest is 66.79 km^2^.

Google Earth images (0.5 m resolution) and 5 m resolution DEMs (provided by the Shaanxi Survey and Mapping Bureau) were used for identifying check dam systems. Google historical images were obtained through Google Earth Pro and SAS software. We downloaded the 19th-level remote sensing images of Suide County with a ground resolution of 0.54 m to extract dam-controlled areas, and the images displayed by Google Earth were taken on 4 March 2021. Google Earth images are provided by professional earth image product and service providers, and the fused images were provided by the Pleiades Neo satellite (Airbus) and SPOT satellite (CNES). Then we used SAS software to mosaic the images and assign them to the WGS84 coordinate system. These images are obtained through resampling, which transforms the resolution from 0.54 to 0.5 m. An appropriate resolution is necessary to achieve accurate object extraction. For the DEM data with a 5 m resolution, resampling facilitates uniformly segmented images. The check dams are clear at this spatial resolution (Figure 2). The use of these images allowed for the identification of dam areas using the deep-learning method. DEMs provide the required topographic features for the elimination of misclassified patches and the improvement of the results.

### 2.2. Overall

**Figure 3:**
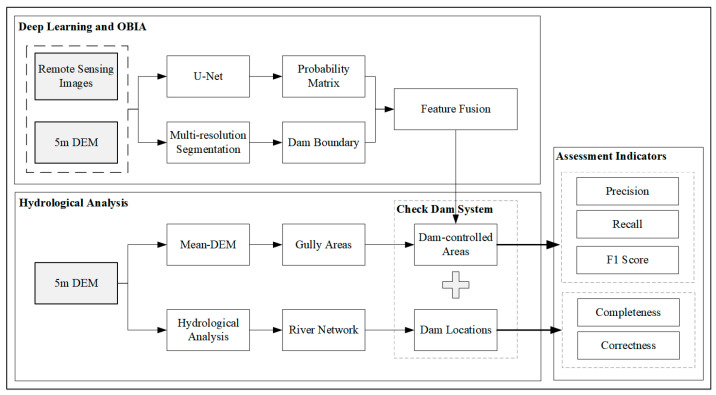
Workflow of the proposed method.

As the Figure 3 shown, the methodology includes two parts: the extraction of dam-controlled areas and the identification of dam locations. First, the OBIA and deep-learning methods were combined to extract dam-controlled areas. The deep-learning technique was employed to determine the probability of pixels that belong to dam-controlled areas in remote sensing images, and the object-oriented multi-resolution segmentation method was used to identify the boundaries of dam-controlled areas from images and DEMs. Following that, the majority voting algorithm was used to combine the results of deep learning and OBIA to obtain the final dam-controlled areas. Then, the dam locations were identified according to the extracted dam-controlled areas and DEMs using the hydrological analysis.

### 2.3. Extracting the Dam-Controlled Areas

#### 2.3.1. Training Datasets

Deep-learning models, like convolutional neural networks (CNN) in image data processing, require more parameters than conventional methods and machine learning; hence, a substantial amount of labeled data is required to train the model to assure accuracy [21]. We prepared the sample labels first using ArcGIS 10.7 software. The samples in this experiment were divided into two classes: the dam class was referred to as the “foreground”, and its complementary class was referred to as the “background” or “non-dam” class. All dam-controlled area polygons were vectorized using ArcGIS 10.7 software, and then the vector data were converted into raster labels using the “Feature to Raster” tool. Next, there were 6 research areas (Figure 2) in Suide County that were selected to construct the training dataset; these areas include dam-controlled areas with a variety of characteristics, and the Jiuyuangou watershed was used as a validation dataset. These datasets were subdivided into small images to meet the input requirements of the deep-learning model, so we set the sample size to 256 × 256 pixels, and a total of 20,000 images were obtained. In this experiment, 1350 (including 315 dam-controlled areas) and 904 images (including 209 dam-controlled areas) with dam-controlled area information were selected as training data and validation data, respectively.

**Figure 4:**
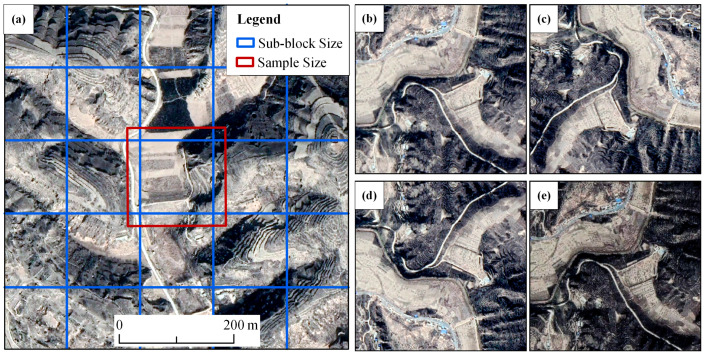
Sample size expansion and data augmentation. (**a**) The sample (red square) had a portion overlapping with its adjacent samples; (**b**–**e**) images of check dams after data augmentation; (**b**) original image; (**c**) rotation 90°; (**d**) horizontal flip; (**e**) sharpen.

The training sample region has a large amount of data, and the rich image features ensure that the model can abstract numerous high-level semantic features, boosting the model’s accuracy and robustness. Because the CNN model’s huge input images may quickly lead to a high number of model parameters and computational complexity, it affects the model’s training and inference efficiency while requiring a considerable amount of internal storage [21]. As a result, images must be segmented into 256 × 256 pixel sub-blocks. The original image was separated into overlapping sub-blocks [22] with a particular overlap rate of 50% (384 × 384 pixels) to maintain the dam-controlled area landscape geometry and texture feature information in the sub-block as much as possible while minimizing the edge effect (Figure 4a). Each overlap area was considered when processing the samples but was discarded during the mosaic process. As Figure 4a shows, the red square represents the sample size, and the blue squares are the sub-blocks used to extract the results. The areas between the red and blue boundaries are discarded.

For deep learning, the amount of data determines the effect of the model to a certain extent, and too little data will lead to overfitting of the model. In the research area, the dam-controlled areas only account for 3.61%, and the size of the sample is small. To overcome this problem, we made full use of the U-Net model’s strengths. The data augmentation enables the neural network to learn more high-level terrain features. Training samples were enhanced approximately three times (3616) by rotating, flipping, and sharpening the original images (Figure 4b–e).

#### 2.3.2. Structure of DCNN

We used a typical deep convolution neural network (DCNN) architecture called U-Net [23] to implement dam-controlled area extraction. The U-Net structure is an end-to-end, fully convolutional neural network architecture, but slightly different from FCN in that the U-Net structure is symmetrical, meaning the U-Net is mainly composed of upsampling and downsampling, and this concatenation structure efficiently improves the classification accuracy of the results through the connection of the contracting path and the expansive path [23]. The U-Net has the benefits of being rapid in operation, capable of yielding more precise segmentation with very few training images, and able to realize multi-scale feature classification of image features via several pooling layers [24].

**Figure 5:**
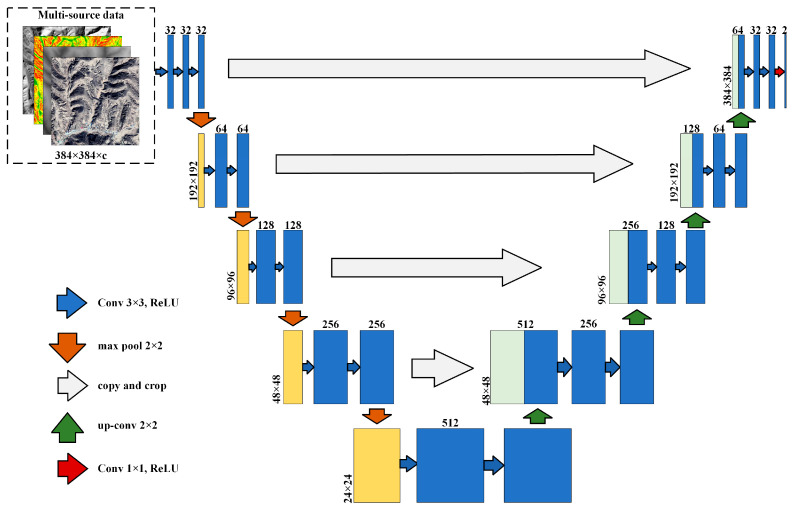
The structure of the U-Net.

Each level of the U-Net structure (Figure 5) is composed of different types and numbers of convolutional layers, pooling layers of fully connected layers. The contracting path (left part of the structure) is an encoder used to extract image features. The size of the input image is 384 × 384 × c (“c” represents the number of multi-source data bands). First, the input image was converted into a 32-dimensional feature map using convolutional operations, and then two convolutional layers and one max pooling layer were repeated. The expansive path can restore the details and position information of the images. At every level in the expansive path, a deconvolution operation is performed to halve the dimension of the feature map before splicing the corresponding feature map from the contracting path, followed by two convolutions. The skip connection helps recover spatial information lost during downsampling, speeds up convergence, and allows very deep networks to be trained [25]. In the final output layer, a 1 × 1 convolution is used to map the feature map obtained in the previous layer into two feature maps: the “dam class” feature map and the “non-dam class” feature map. The feature map of the “dam class” represents the score of each pixel corresponding to the dam class.

The dataset in this case consisted of topographic images. Due to the complexity of terrain texture, the conventional U-Net structure has difficulties capturing terrain features. Images, for instance, can convey a landform’s texture features but not its surface elevation information. Multi-source data produce significantly higher prediction accuracy than a single data source [26]. Therefore, we simultaneously input multi-source data into the U-Net model to compensate for these shortcomings. One longitudinal study [27] found that integrating remote sensing images, the digital elevation model (DEM), and its derivatives into the machine learning model yields a much-improved detection result. As shown in Figure 5, the DEM, slope, hillshade, and remote sensing images were merged as the data source and input into the model. 

#### 2.3.3. Multi-Resolution Segmentation

**Figure 6:**
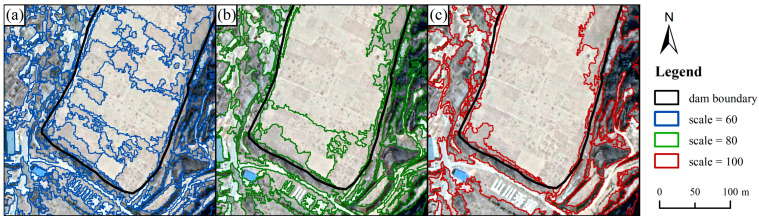
Multi-resolution segmentation results with different scale parameters. (**a**) scale = 60; (**b**) scale = 80; (**c**) scale = 100 (their shape and compactness parameters are all 0.1 and 0.5, respectively).

The deep-learning structure (U-Net) provided a probability image for determining check dam pixels. However, the accurate check dam boundaries are hard to determine with the probability image. We used the multi-resolution segmentation (MRS) [28] approach to identify the boundaries of dam-controlled areas. Scale, shape, and compactness are the three main segmentation factors that influence the outcomes of image segmentation. Figure 6 shows the segmentation results for various segmentation scales. By adjusting the scale parameter values while controlling the shape and compactness parameters, it is clear that the smaller the scale parameter, the finer the dam-controlled area is segmented, but the subsequent workload increases. Conversely, a value that is too large will result in an incorrect classification of the more complex areas of the landscape. Using the ESP2 (estimation of scale parameter 2) tool [29], the potential optimal segmentation scale parameter was obtained, and we selected a scale parameter of 100 (Figure 6c) for the object-oriented multi-resolution segmentation in this study area. Based on previous studies [14,23,30], a shape parameter of 0.1 and compactness of 0.5 was suitable for terrain segmentation. Figure 6 shows the segmentation results using these parameters, which are superior to those of other parameters.

#### 2.3.4. Feature Fusion

The OBIA and deep-learning methods have their advantages. The deep-learning method assigned a probability value of the dam-controlled area for each pixel, while the OBIA provided patches with internal homogeneity. Here, we used majority voting to combine the results of deep learning and OBIA and then determined the dam-controlled areas. First, the patches provided by the OBIA method were overlaid with the probability image. The majority value of the probability image in each patch was calculated and assigned as the patch’s value. Second, referring to the previous experimental results [14], a 0.5 threshold was used to determine the dam patches. If the patches’ value is greater than 0.5, they are regarded as potential dam-controlled areas. Moreover, given that check dams were generally located in gully areas, we used the gully areas to filter noise. The gully areas were extracted from the 5 m DEMs by the multi-directional hillshading method [31,32,33]. Then, the potential dam patches were overlaid with gully areas. Only the patches located in the gully areas were regarded as true dam-controlled areas.

### 2.4. Extracting Dam Locations

**Figure 7:**
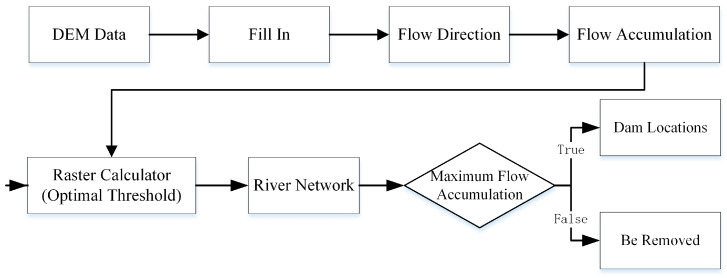
Flow diagram of extracting dam locations.

A check dam is generally built at the watershed or sub-watershed outlet, where the flow accumulation is the largest. Therefore, after extracting the dam-controlled areas, the location of the check dam can be determined by using the hydrological analysis approach to identify the area of greatest flow accumulation (Figure 7). First, we used the traditional hydrological analysis [34,35] (including fill, flow direction, and flow accumulation) to determine the river network. Using the “extract by mask” tool in ArcGIS 10.7, the flow accumulation raster of each dam-controlled area was obtained. Then, we calculated the maximum flow accumulation of each dam-controlled area and put that information together with the river network to determine the final dam locations.

### 2.5. Accuracy Assessment

#### 2.5.1. Accuracy of Dam-Controlled Area Extraction

To validate the accuracy of the extracted dam-controlled areas, we manually interpreted the dam areas from remote sensing images and considered these areas as the reference data. Then we calculated the area errors of each extracted dam (the area difference between the dam extracted by the proposed method and the visual interpretation of dams). 

The relative bias (${\mathrm{RB}}_{i}$) [36] indicator was adopted to quantify the area errors for each dam, and the closer the index is to 0, the smaller the error of the area of the check dam estimated by the proposed method is. A negative value indicates that the measured area of the check dam is smaller than the area estimated from the remote sensing image, and the opposite is true for a positive value. From the spatial perspective, the ${\mathrm{RB}}_{i}$ spatial distribution could show the performance of the proposed method in different locations of the watershed. The correlation coefficient (CC) [37] for the overall performance is used to verify whether there is a good linear correlation between the results of the proposed method and the actual areas. The formulas are as follows:

(1) $$\mathrm{CC}=\frac{\sum_{i=1}^{n}\left(x_{i}-\bar{x}\right)\left(y_{i}-\bar{y}\right)}{{\sqrt{\sum_{i=1}^{n}{\left(x_{i}-\bar{x}\right)}^{2}\sum_{i=1}^{n}{\left(y_{i}-\bar{y}\right)}^{2}}}^{}}$$

(2) $${\mathrm{RB}}_{i}=\frac{\sum_{i=1}^{n}\left(x_{i}-y_{i}\right)}{\sum_{i=1}^{n}y_{i}}$$

where x represents the actual dam-controlled area (the reference data), y is the area extracted by the proposed method, and n is the total area of dam-controlled areas.

Moreover, in this paper, dam-controlled area identification belongs to semantic segmentation classification. Precision, recall, and F1-score [38] are commonly used as evaluation indicators in the binary classification of remote sensing images. These indicators are general evaluation indicators to evaluate the merits of the model. Precision represents the ratio of the area correctly predicted as dams to all the area predicted as dams, demonstrating how well the dam areas were extracted correctly. The recall is the proportion of the correctly predicted dam-controlled area to the actual dam-controlled area, which reflects how much the dam areas have been completely detected. The F1 score shows the overall assessment of precision and recall.

(3) $$\mathrm{precision}=\frac{\mathrm{TP}}{\mathrm{TP}+\mathrm{FP}}$$

(4) $$\mathrm{recall}=\frac{\mathrm{TP}}{\mathrm{TP}+\mathrm{FN}}$$

(5) $$F1\;\mathrm{score}=\frac{2\times \mathrm{precision}\times \mathrm{recall}}{\mathrm{precision}+\mathrm{recall}}$$

TP, FP,TN, and FN represent the areas of the true positive, false positive, true negative, and false negative pixels, respectively. 

#### 2.5.2. Accuracy of Dam Location Extraction

Dam locations visually interpreted from the Google Earth remote sensing images were considered the reference data. We used the window analysis method (the window size is 11 × 11) to calculate the maximum flow accumulation raster within each dam-controlled area as the final dam locations. The dams falling within the buffer zone of reference data were considered “correct detections”, while those falling outside the zone were “incorrect detections”. Given that the DEM data resolution was 5 m, we set the buffer radius to 20 m. Then we calculated the correctness and completeness [39]:

(6) $$A_{c}=E_{c}/E_{t}$$

(7) $$C_{r}=E_{c}/P_{c}$$

where $A_{c}$ is correctness, $C_{r}$ is completeness, $E_{c}$ is the number of correctly extracted dam locations, $E_{t}$ is the total number of extracted dam locations, and $P_{c}$ is the number of visually interpreted dam locations. 

The correctness represents the percentage of the dam location identified by the proposed method that lies within the buffer around the reference location. The completeness shows that a percentage of the dam location extracted by the proposed method lies within the buffer.

## 3. Results and Discussion

### 3.1. Dam Areas and Locations

#### 3.1.1. Results of Dam-Controlled Area Extraction

**Figure 8:**
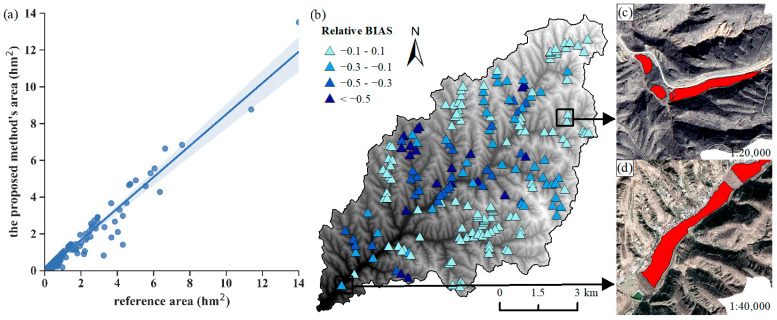
(**a**) Correlation between the dam-controlled areas from the proposed method and the reference areas; (**b**) spatial distribution characteristics of relative bias; (**c**,**d**) enlarged images of dam-controlled areas.

According to the result of manual visual interpretation from high-resolution remote sensing images, we selected 209 dam-controlled areas in the study area. As shown in Figure 8a, a particularly good linear fit exists among the areas derived from remote sensing images with the visual interpretation method and the areas extracted by the proposed method. The correlation coefficient is 0.97, which illustrates that the size of the dam-controlled area is similar in the same place.

Among the 209 extracted dam-controlled areas, there are 83 areas whose bias value is close to zero, indicating that the area extracted by the proposed method is similar to the actual one. There are 123 dam elements (roughly 60% of total dams) with bias values in the interval [−1, 0], showing that the proposed method tends to underestimate the actual dam-controlled areas. According to Figure 8b, the errors in the upstream of the watershed and the tributaries in the southeast of the watershed are relatively small, with the relative error in the interval [−0.1, 0.1]. There are certain dam-controlled areas with higher errors in the midstream of the watershed, and since dams located in the midstream of the watershed were constructed relatively earlier, it could be inferred that the error distribution could be related to the age of the dams. The Jiuyuangou watershed is in the semi-arid temperate zone, and its average annual precipitation has been 469 mm for many years. The rain is concentrated in the period from July to September, with the majority falling as heavy rain. The older check dam systems were likely washed away by the heavy rain or filled with deposited sediment; thus, damaged or filled check dam systems may lead to higher errors in the extraction of dam-controlled areas using the deep-learning method [37].

To further analyze the overall extraction accuracy, we calculated the precision, recall, and F1 score by generating the confusion matrix. The precision and recall are 98.56% and 84.20%, respectively (Table 1). The high precision and relatively low recall are possible because the training data only account for 3.61% of the whole study area. The F1 score is a weighted average score of the true positive (recall) and precision, so it is applied to comprehensively judge the overall classification performance of the deep learning and the OBIA method, and the value of the F1 score is 89.70%, demonstrating the favorable extraction result of the proposed model.

#### 3.1.2. Results of Dam Location Extraction

**Figure 9:**
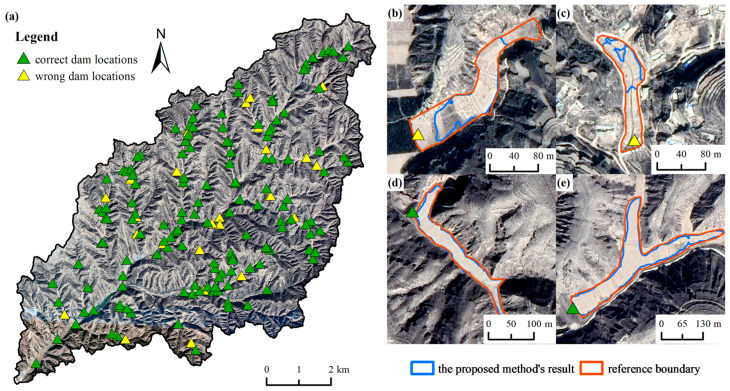
(**a**) The distribution of extracted dam locations; (**b**,**c**) enlarged images of small check dam systems; (**d**,**e**) enlarged images of medium and large check dam systems.

According to the labeled dam-controlled areas, we selected 182 dam locations from the total dam locations extracted by visual interpretation for experimental analysis. We extracted 172 dam locations using hydrological analysis and deep-learning methods, with 147 of them correctly extracted. The completeness and correctness rates are 94.51% and 80.77%, respectively. Figure 9a shows that many wrongly identified dam locations are small check dams located in tributaries; moreover, the extraction result of large and medium dams is better than that of small dams. The accuracy of dam locations highly relies on the extraction of dam-controlled areas. Figure 9b,c are examples of incorrectly extracted small check dams, and the extracted dam-controlled areas are quite different from the actual areas; thus, identifying the dam locations based on the inaccurately extracted dam-controlled areas can lead to an unsatisfactory extraction result for dam locations. In contrast, the extraction results of medium and large dams are generally better (Figure 9d,e), and the dam locations are closer to the actual ones.

### 3.2. Comparison with the OBIA Method

**Figure 10:**
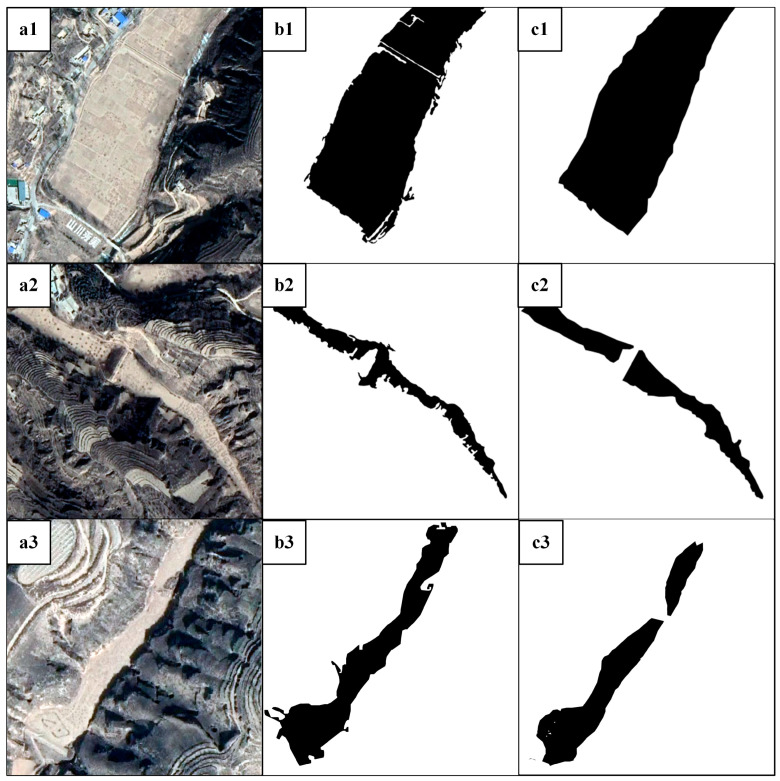
Results of the two extraction methods compared in terms of shape: (**a1**–**a3**) Google Earth remote sensing images; (**b1**–**b3**) the OBIA method’s results; (**c1**–**c3**) the proposed method’s results.

We compared the proposed method with the traditional OBIA method [40]. The OBIA method obtains potential dam-controlled areas using multi-resolution segmentation and aggregates them using nearest-neighbor classification. In Figure 10, the remote sensing image data, the corresponding OBIA extraction results, and the proposed method extraction results are respectively displayed. Due to the phenomenon of the same object with different spectra or different objects with the same spectra, the ground patches extracted by the traditional OBIA method are fragmented, have unclear edges, and contain small holes. When the OBIA provides the ground object boundary constraint, deep learning can yield more reliable extraction results, the extracted edges are smoother and distinct, there are fewer holes within, and the edges more closely resemble the actual edges on the ground. The results of dam-controlled area extraction using the method of deep learning combined with OBIA proposed in this paper show that our model can effectively extract dam-controlled areas in the Jiuyuangou watershed.

In terms of the shape of the extracted dam-controlled area, the proposed method is better than the traditional OBIA method. To better compare the performance of overall extraction accuracy between the two methods, we also applied the confusion matrix to evaluate the OBIA extraction result, then compared those three indicators of the two methods. As can be seen from Table 1, the proposed method surpassed the OBIA method in all overall accuracy assessment indicators. The precision of the proposed method is much higher than that of the OBIA method, with a large gap of 14.96%, and the recall of the proposed method is nearly 11% higher than that of the OBIA method with nearest-neighbor classification; the latter incorrectly classifies the terraces and mounds as dam-controlled areas. The proposed method’s F1 score increases by 12.7%. In general, the dam-controlled area extraction method based on deep learning and OBIA is much better than the object-based image analysis method with the nearest-neighbor classification method. When compared to the traditional OBIA method, the proposed extraction method produced better results and significantly improved accuracy in high-resolution image classification research, indicating higher feasibility and applicability.

### 3.3. Distribution Characteristics of the Check Dam System

**Figure 11:**
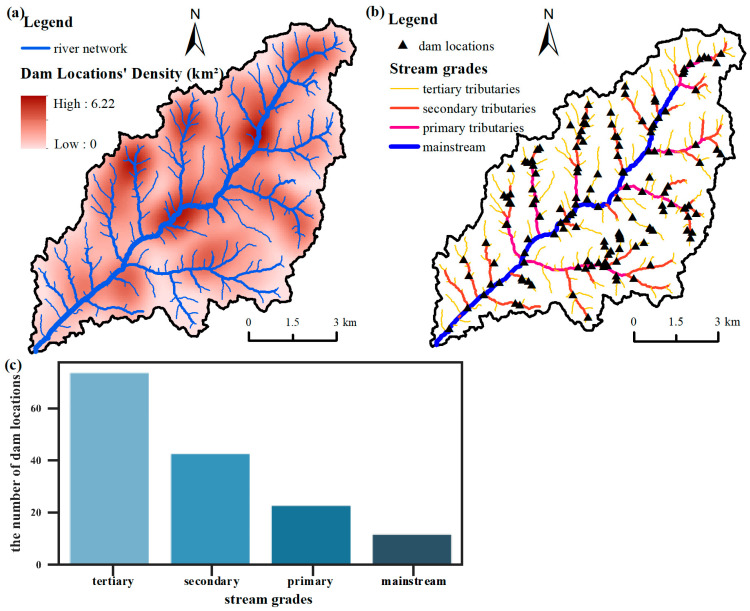
Spatial distribution of check dam systems: (**a**) dam location density; (**b**) distribution of check dams in different stream grades; (**c**) histogram of check dams in different stream grades.

To investigate the geographical distribution of the check dams in the watershed, the point density of the check dams (Figure 11a) was calculated. The density of check dam systems in the watershed ranges from 0 to 6.22 per square kilometer. The upstream and downstream portions of the study area are narrow, while the midstream is wider, and the watershed system presents a typical dendritic shape. The area of the midstream is extensive, including many mainstreams and tributaries, so a large number of check dams are built in the channels. The mainstream in the midstream and the part on its left side are dense areas with large amounts of check dams. Combined with Figure 11b, it can be seen that there are fewer tributaries on the left side of the mainstream than on the right side, indicating that the erosion degree on the right side is more serious than that on the left side, which coincides with the phenomenon that the number of check dams on the right side of the mainstream is greater than that on the other side.

To further analyze the distribution of the dam system, we classified the stream grades in the study and calculated the number of dam locations distributed in each stream grade. Figure 11c shows that the number of check dam locations distributed in the mainstream, primary tributaries, secondary tributaries, and tertiary tributaries is increasing, and the check dam system’s control capacity over the entire watershed is gradually improving. It is noticeable that the rapid increase in the number of check dams from the tertiary tributaries to the primary tributaries can also reflect the high development degree of the watershed and serious soil erosion in the study area.

## 4. Conclusions

An effective method combing deep learning and hydrological analysis was proposed in this paper for identifying check dam systems from remote sensing images and DEMs. First, deep learning was used to calculate the probability that each pixel belongs to the dam-controlled area, and multi-resolution segmentation was applied in this step to generate objects with precise boundaries. Following that, the majority voting algorithm integrated deep learning and OBIA results to obtain the final dam-controlled areas. In this study, the use of multi-resolution segmentation can effectively avoid the influence of impulse noise, and the U-Net architecture achieves good performance on check dam extraction thanks to data augmentation with elastic deformations. The Jiuyuangou watershed was chosen as the study area to validate the proposed method, and this method yields good extraction results with high accuracy. The correlation between dam-controlled areas and actual areas is 0.97, and the relative bias indicates that the proposed method tends to underestimate the dam-controlled areas. The second step is identifying the dam location based on the extracted dam-controlled areas. The spatial relationship between the dam-controlled area and the river network and the maximum flow accumulation were used to determine the dam locations. The completeness of dam locations is 94.51%; however, the extraction accuracy of dam locations is highly dependent on the extraction of dam-controlled areas. We also compared the proposed method with the traditional OBIA method using nearest-neighbor classification. In identifying the boundaries and dam-controlled areas, the proposed method outperformed the OBIA method; precision, recall, and F1 score of the proposed method are 14.60%, 11.20%, and 12.70% higher than the latter, respectively.

The proposed approach can be applied to extract check dam systems and provide basic data for maintaining dam infrastructure, optimizing the spatial configuration of dam systems, and calculating the amount of silt. By investigating the spatial distribution characteristics of the check dam system, we have discovered that the check dam systems have aggregation areas within the watershed, with the number of dam locations on the right side of the mainstream being greater than on the left side; and the spatial distribution is related to stream grades: as tributary grades increase, so does the number of check dam systems.

Following are a few points that can be further studied in theory and practice:

1. The proposed method is restricted by vegetation. In the Yellow River Basin, many check dams are covered with crops or vegetation. In this study, remote sensing images shot in winter were selected to avoid the influence of vegetation. However, if images taken in summertime are used, dams are covered by different kinds of vegetation, which makes it difficult to identify the dams in the images.

2. The accuracy of determining the dam location depends on the result of identifying the dam-controlled area. To overcome the difficulty of directly extracting the dam location, we used the hydrological analysis to obtain the location from the dam-controlled areas. While this strategy is able to detect most of the dam locations, it is challenging to identify all of the locations.

In the follow-up research, multi-spectral remote sensing images can be taken into consideration to improve the model effect. At the same time, multiple deep-learning networks should be compared and analyzed to find the best network to identify the check dam system or to improve the existing networks based on the check dam’s spectral and morphological characteristics. 

## Figures and Tables

**Table 1:** Results of the two dam-controlled area extraction methods.

	Proposed Method	OBIA Method
Confusion matrix basic indicators (km^2^)		
TP	2.06	1.78
TN	66.72	66.45
FP	0.03	0.34
FN	0.13	0.72
Overall accuracy assessment indicators		
Precision	98.56%	83.96%
Recall	82.40%	71.20%
F1 Score	89.76%	77.06%

## Data Availability

The data that support the findings of this research are available from the author upon reasonable request.

## References

[1] Marsh W.M. (2005). Landscape Planning: Environmental Applications.

[2] Wang S., Fu B., Piao S., Lü Y., Ciais P., Feng X., Wang Y. (2016). Reduced Sediment Transport in the Yellow River Due to Anthropogenic Changes. Nat. Geosci..

[3] Gilichinsky M., Peled A. (2013). Detection of Discrepancies in Land-Use Classification Using Multitemporal Ikonos Satellite Data. Int. Arch. Photogramm. Remote Sens. Spat. Inf. Sci..

[4] Dai W., Qian W., Liu A., Wang C., Yang X., Hu G., Tang G. (2022). Monitoring and modeling sediment transport in space in small loess catchments using UAV-SfM photogrammetry. CATENA.

[5] Dai W., Hu G., Yang X., Yang X.W., Cheng Y., Xiong L., Strobl J., Tang G.A. (2020). Identifying ephemeral gullies from high-resolution images and DEMs using flow-directional detection. J. Mt. Sci..

[6] Blaschke T., Hay G.J., Kelly M., Lang S., Hofmann P., Addink E., Queiroz Feitosa R., van der Meer F., van der Werff H., van Coillie F. (2014). Geographic Object-Based Image Analysis—Towards a New Paradigm. ISPRS J. Photogramm. Remote Sens..

[7] Dai W., Na J., Huang N., Hu G., Yang X., Tang G.A., Xiong L., Li F. (2019). Integrated edge detection and terrain analysis for agricultural terrace delineation from remote sensing images. Int. J. Geogr. Inf. Sci..

[8] Yang X., Dai W., Tang G., Li M. (2017). Deriving Ephemeral Gullies from VHR Image in Loess Hilly Areas through Directional Edge Detection. ISPRS Int. J. Geo-Inf..

[9] Blaschke T. (2010). Object Based Image Analysis for Remote Sensing. ISPRS J. Photogramm. Remote Sens..

[10] Wei H., Xiong L., Zhao F., Tang G., Lane S. (2022). Large-Scale Spatial Variability in Loess Landforms and Their Evolution, Luohe River Basin, Chinese Loess Plateau. Geomorphology.

[11] Li X., Shao G. (2014). Object-Based Land-Cover Mapping with High Resolution Aerial Photography at a County Scale in Midwestern USA. Remote Sens..

[12] LeCun Y., Bengio Y., Hinton G. (2015). Deep Learning. Nature.

[13] Stanislawski L.V., Shavers E.J., Wang S., Jiang Z., Usery E.L., Moak E., Duffy A., Schott J. (2021). Extensibility of U-Net Neural Network Model for Hydrographic Feature Extraction and Implications for Hydrologic Modeling. Remote Sens..

[14] Li S., Xiong L., Hu G., Dang W., Tang G., Strobl J. (2021). Extracting Check Dam Areas from High-resolution Imagery Based on the Integration of Object-based Image Analysis and Deep Learning. Land Degrad. Dev..

[15] Tong X.-Y., Xia G.-S., Lu Q., Shen H., Li S., You S., Zhang L. (2020). Land-Cover Classification with High-Resolution Remote Sensing Images Using Transferable Deep Models. Remote Sens. Environ..

[16] Pereira V., Cabral F., Pereira L., Fukai H. (2021). Implementation of U-Net Deep Learning Framework for Road and Road Line Segmentation. Timorese Acad. J. Sci. Technol..

[17] Yan C., Fan X., Fan J., Wang N. (2022). Improved U-Net Remote Sensing Classification Algorithm Based on Multi-Feature Fusion Perception. Remote Sens..

[18] Sun L., Guo H., Chen Z., Yin Z., Feng H., Wu S., Siddique K.H.M. (2023). Check Dam Extraction from Remote Sensing Images Using Deep Learning and Geospatial Analysis: A Case Study in the Yanhe River Basin of the Loess Plateau, China. J. Arid Land.

[19] Zhao W., Du S. (2016). Learning Multiscale and Deep Representations for Classifying Remotely Sensed Imagery. ISPRS J. Photogramm. Remote Sens..

[20] Chen Y., Ming D., Lv X. (2019). Superpixel Based Land Cover Classification of VHR Satellite Image Combining Multi-Scale CNN and Scale Parameter Estimation. Earth Sci. Inform..

[21] Krizhevsky A., Sutskever I., Hinton G.E. (2017). ImageNet Classification with Deep Convolutional Neural Networks. Commun. ACM.

[22] Huang L., Liu L., Jiang L., Zhang T. (2018). Automatic Mapping of Thermokarst Landforms from Remote Sensing Images Using Deep Learning: A Case Study in the Northeastern Tibetan Plateau. Remote Sens..

[23] Ronneberger O., Fischer P., Brox T., Navab N., Hornegger J., Wells W.M., Frangi A.F. (2015). U-Net: Convolutional Networks for Biomedical Image Segmentation. Proceedings of the Medical Image Computing and Computer-Assisted Intervention—MICCAI 2015.

[24] Li C., Fu L., Zhu Q., Zhu J., Fang Z., Xie Y., Guo Y., Gong Y. (2021). Attention Enhanced U-Net for Building Extraction from Farmland Based on Google and WorldView-2 Remote Sensing Images. Remote Sens..

[25] Drozdzal M., Vorontsov E., Chartrand G., Kadoury S., Pal C., Carneiro G., Mateus D., Peter L., Bradley A., Tavares J.M.R.S., Belagiannis V., Papa J.P., Nascimento J.C., Loog M., Lu Z. (2016). The Importance of Skip Connections in Biomedical Image Segmentation. Proceedings of the Deep Learning and Data Labeling for Medical Applications.

[26] Xiong L., Li S., Tang G., Strobl J. (2022). Geomorphometry and terrain analysis: Data, methods, platforms and applications. Earth-Sci. Rev..

[27] Wang S., Li W. (2021). GeoAI in Terrain Analysis: Enabling Multi-Source Deep Learning and Data Fusion for Natural Feature Detection. Comput. Environ. Urban Syst..

[28] Baatz M., Schape A. (2000). Multiresolution Segmentation: An Optimization Approach for High Quality Multi-Scale Image Segmentation. Angewandte Geographische Informationsverarbeitung.

[29] Drăguţ L., Csillik O., Eisank C., Tiede D. (2014). Automated Parameterisation for Multi-Scale Image Segmentation on Multiple Layers. ISPRS J. Photogramm. Remote Sens..

[30] Bhowmick S., Nagarajaiah S., Veeraraghavan A. (2020). Vision and Deep Learning-Based Algorithms to Detect and Quantify Cracks on Concrete Surfaces from UAV Videos. Sensors.

[31] Dai W., Yang X., Na J., Li J., Brus D., Xiong L., Tang G., Huang X. (2019). Effects of DEM Resolution on the Accuracy of Gully Maps in Loess Hilly Areas. CATENA.

[32] Na J., Yang X., Dai W., Li M., Xiong L., Zhu R., Tang G. (2018). Bidirectional DEM Relief Shading Method for Extraction of Gully Shoulder Line in Loess Tableland Area. Phys. Geogr..

[33] Yang X., Li M., Na J., Liu K. (2017). Gully Boundary Extraction Based on Multidirectional Hill-Shading from High-Resolution DEMs. Trans. GIS.

[34] Burrough P.A., McDonnell R.A., Lloyd C.D. (2015). Principles of Geographical Information Systems.

[35] Wilson J., Gallant J., Hutchinson M.F. (2000). Future Directions for Terrain Analysis.

[36] Tian Y., Zhong Z., Ordonez V., Kaiser G., Ray B. (2020). Testing DNN Image Classifiers for Confusion & Bias Errors. Proceedings of the ACM/IEEE 42nd International Conference on Software Engineering.

[37] Tian P., Zhao G., Mu X., Wang F., Gao P., Mi Z. (2013). Check Dam Identification Using Multisource Data and Their Effects on Streamflow and Sediment Load in a Chinese Loess Plateau Catchment. JARS.

[38] Mäkinen V., Oksanen J., Sarjakoski T. (2019). Automatic Determination of Stream Networks from DEMs by Using Road Network Data to Locate Culverts. Int. J. Geogr. Inf. Sci..

[39] WDai W., Hu G., Huang N., Zhang P., Yang X., Tang G. (2019). A contour-directional detection for deriving terrace ridge from open source images and digital elevation models. IEEE Access.

[40] Chen X. (2020). Information Extraction and Feature Analysis of Sediment Retention Dam in Loess Plateau Based on High Precision DEM. Master’s Thesis.

